# Vulvar mucinous cyst mimicking common lesions with concurrent multiple bartholin cysts in a reproductive-age woman: A rare case report and review of literature

**DOI:** 10.18632/oncoscience.630

**Published:** 2025-10-09

**Authors:** Naina Kumar, Immanuel Pradeep, Banka Sai Swetha, Pooja T. Rathod

**Affiliations:** ^1^Department of Obstetrics and Gynecology, All India Institute of Medical Sciences, Bibinagar 508126, Hyderabad, Telangana, India; ^2^Department of Pathology and Lab Medicine, All India Institute of Medical Sciences, Bibinagar 508126, Hyderabad, Telangana, India

**Keywords:** bartholin cyst, gartner’s cyst, lipoma, mucinous cyst, vulva

## Abstract

Introduction: Vulvovaginal cysts are typically benign and asymptomatic, often going unnoticed during routine clinical evaluations. However, rare variants, such as vulvar mucinous cysts, can present atypically, sometimes mimicking more common lesions, like lipomas. Bartholin gland cysts, though common, may coexist with other unusual vulvar cysts, making accurate diagnosis essential for appropriate management. Case Report: A 36-year-old multiparous woman presented with lower abdominal and back pain, accompanied by a single episode of prolonged menstrual bleeding. On local examination, a soft, pedunculated, asymptomatic mass measuring 3 × 4 cm was observed on the left labia majora, clinically resembling a vulvar lipoma. In addition, multiple smaller, non-tender cystic lesions were noted along the inner surface of the left labia minora. Surgical excision of all lesions was performed. Histopathological evaluation identified the labial mass as a mucinous vulvar cyst and the smaller lesions as multiple Bartholin gland cysts, with no evidence of atypia. The postoperative course was uneventful, and the patient was discharged in stable condition with advice to follow up after the next menstrual cycle. Conclusion: This case emphasizes the importance of considering rare vulvar mucinous cysts in the differential diagnosis of asymptomatic vulvar masses. Coexistence with multiple Bartholin cysts adds to the diagnostic complexity. Surgical excision not only provides a definitive diagnosis but also prevents future complications. Histopathological evaluation remains crucial for accurate classification and guiding follow-up.

## INTRODUCTION

Various benign cystic lesions can arise in the vulvar region, and they are relatively common among adult women [1, 2]. Among these, vulvar mucinous cysts are rare, benign, and noninvasive [3]. The etiology of vulvar masses can be broadly categorized into two main origins: embryonic and non-embryonic. Lesions of embryonic origin are more frequently encountered and include cysts derived from remnants of the Müllerian and mesonephric (Gartner’s) ducts, the urogenital sinus, and other embryologic structures. These comprise Müllerian cysts, Gartner’s duct cysts, Bartholin’s duct cysts, vestibular mucous cysts, Skene’s duct cysts, and canal of Nuck cysts [3–5].

Non-embryonic cysts include epidermal inclusion cysts, vaginal endometriosis, and vaginitis emphysematosa [3–5]. Although there is no universally accepted clinical classification for benign vulvar tumors, they are often categorized based on morphology into mucosal, cystic, or solid types [6]. Compared to other vulvar lesions, mucosal cysts are relatively uncommon, and their precise embryologic origins remain a topic of ongoing debate [3].

Vulvovaginal cysts are reported in approximately 1 in 200 women; however, their true prevalence is likely underestimated due to the asymptomatic nature of many cases [5]. Additionally, diagnostic ambiguity arises from the overlap in anatomical and pathological expertise among gynecologists, urologists, surgeons, and dermatologists. While many of these cysts remain clinically silent, some can progressively enlarge and present with a wide spectrum of histopathological findings [7].

The present case highlights a rare mucinous cyst of the vulva, clinically mimicking a vulvar lipoma, accompanied by multiple Bartholin cysts arising from the left vulvo-vaginal region in a reproductive-aged woman.

## CASE REPORT

A 36-year-old woman, para two with one living child (P2L1), presented to the Gynecology Outpatient Department with complaints of lower abdominal and back pain persisting for 1–2 months, along with her first episode of prolonged menstrual bleeding, lasting 10–12 days. There was no history of dysmenorrhea, dyspareunia, vulvodynia, or urinary or bowel disturbances. Her menstrual cycles had previously been regular, occurring every 28–30 days, with bleeding lasting 4–5 days. Her last menstrual period was on May 8, 2025.

She had a known history of hypothyroidism for the past three years and was on regular medication (Thyronorm 25 μg). There was no personal history of diabetes, hypertension, or other comorbidities, and no family history of malignancy.

**Figure 1:**
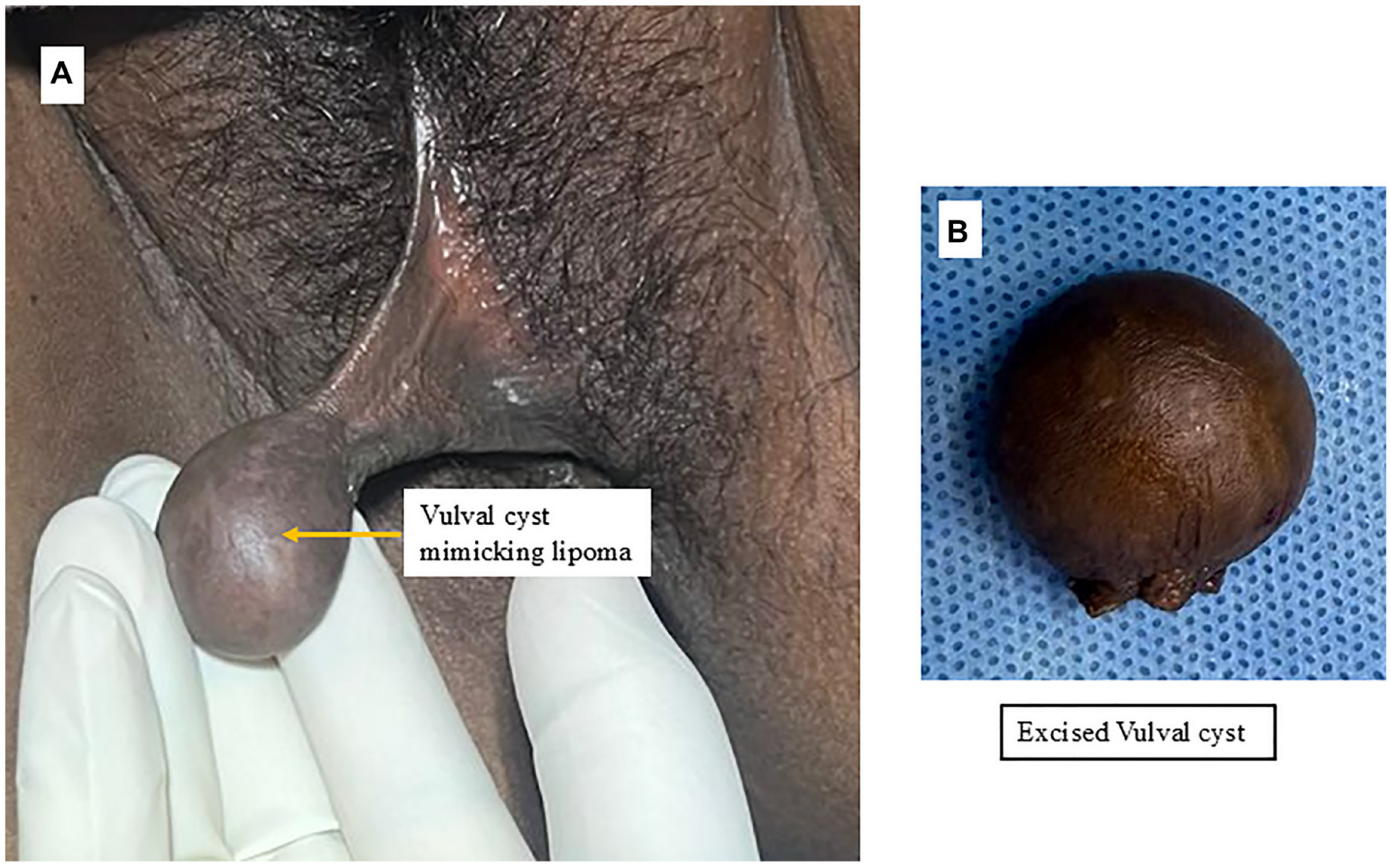
(**A**) Vulval cyst arising from the left labia majora with a broad pedicle. (**B**) The excised vulval cyst covered with skin.

**Figure 2:**
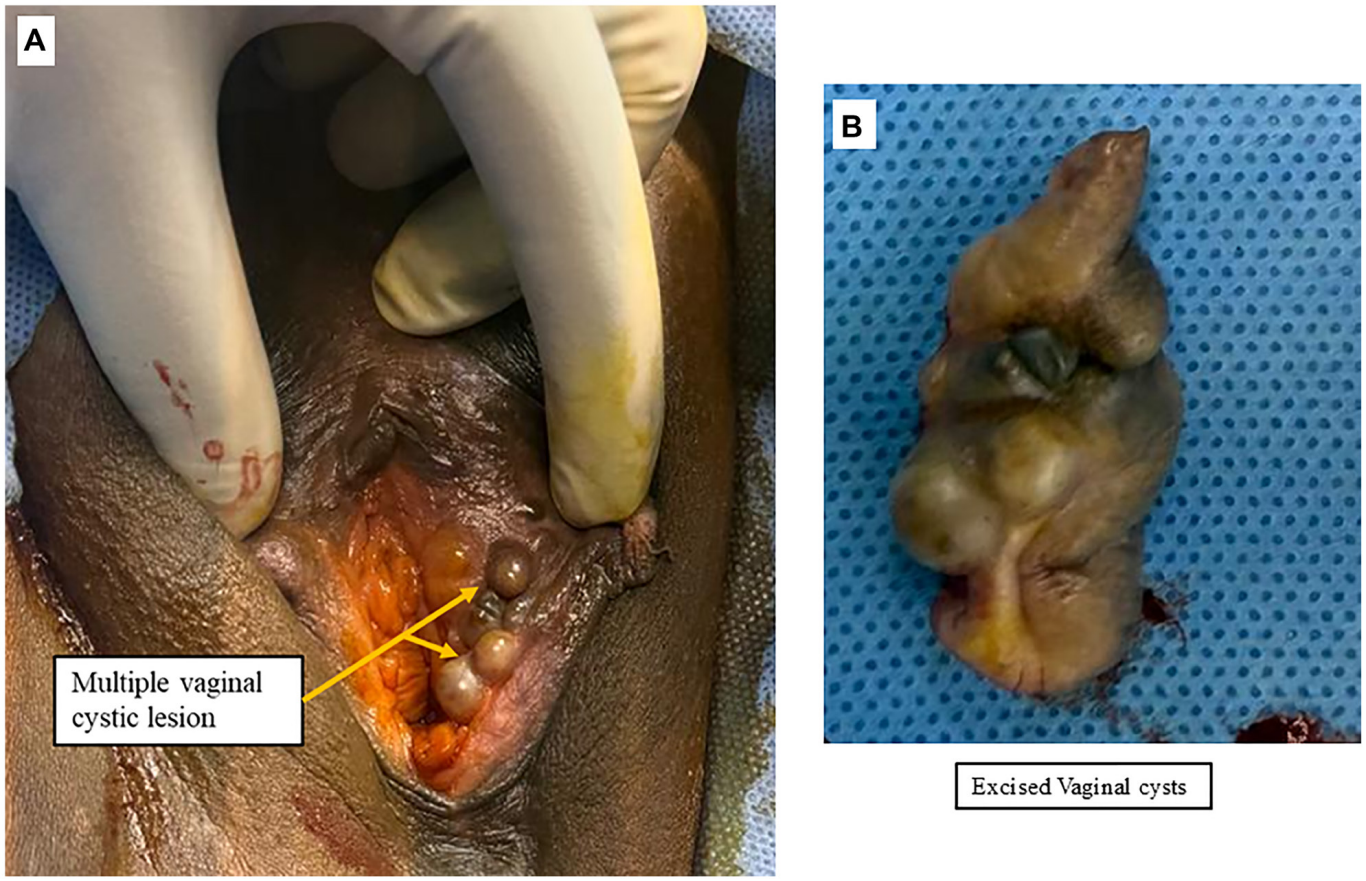
(**A**) Multiple vaginal cysts present on the entire length of the inner margin of labia minora. (**B**) The wide excision of vaginal cysts along with the 1 cm margin of mucosa around it.

On general examination, she was of average build. Abdominal examination was unremarkable, with no tenderness, palpable mass, or organomegaly. Local examination revealed normal pubic hair and a large, pedunculated mass (3 × 4 cm) arising from the left labia majora. It was soft, cystic, and clinically resembled a vulvar lipoma (Figure 1A, 1B). It was asymptomatic. Additionally, multiple non-tender, cystic lesions were observed on the inner aspect of the left labia minora along the vaginal mucosa (Figure 2A). These were also asymptomatic and detected incidentally on examination. Speculum examination revealed a healthy cervix. On bimanual pelvic examination, the uterus was multiparous in size, anteverted, mobile, with free bilateral fornices.

### Investigations

Pelvic ultrasonography showed a normal-sized uterus (8.2 × 4.8 × 4 cm) with an endometrial thickness of 9 mm. Both ovaries appeared normal. Routine laboratory investigations—including complete blood count, liver and renal function tests, blood glucose, and viral markers (HIV, HBsAg, HCV)—were within normal limits. Cervical cytology via Pap smear revealed no intraepithelial lesion.

### Surgical management and histopathological findings

The patient was planned for surgical excision of the vulvovaginal cysts along with endometrial biopsy. A large, cystic, pedunculated mass with an intact skin covering was excised from the mid-left labia majora after clamping and cutting the pedicle at its base (Figure 1B). The vaginal cystic lesions on the left labia minora were excised with a 1 cm margin of adjacent vaginal mucosa (Figure 2B). Hemostasis was achieved, and the surgical wound was closed using 2–0 Vicryl sutures. Endometrial curettage was also performed for histopathological analysis.

Gross examination of the vulvar lesion revealed a skin-covered mass measuring 3.8 × 2.4 × 2.8 cm with a bosselated surface and a cystic cavity filled with mucoid material; cyst wall thickness was 0.2 cm. The excised vaginal lesions measured 3.5 × 2 × 1 cm in total, with the largest cystic space measuring 1.2 × 0.8 cm on cut section, all containing mucoid fluid.

**Figure 3:**
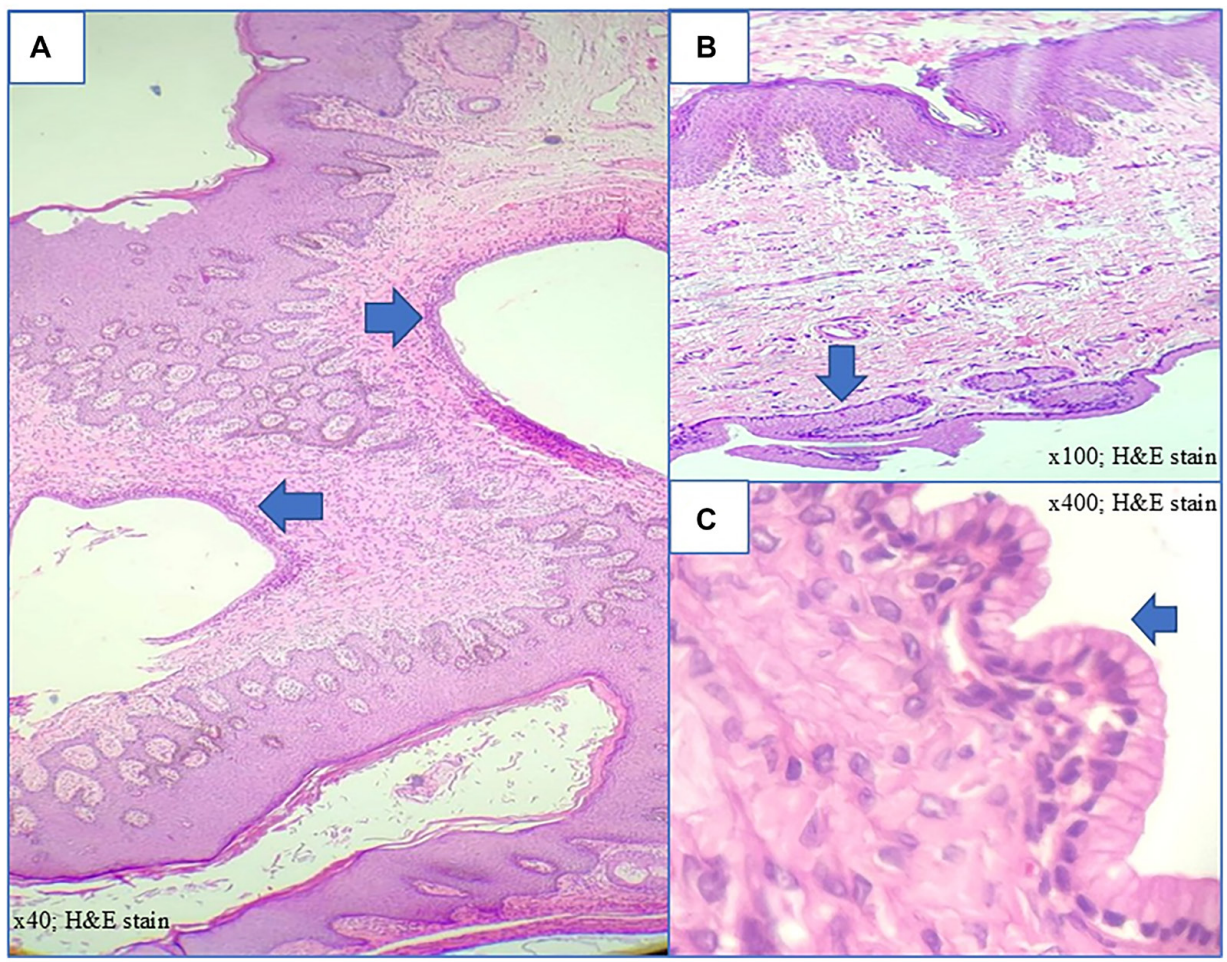
(**A**) Vulval cysts lined by mucinous columnar epithelium (×40; H&E stain) suggestive of mucinous cyst. (**B**) Vaginal Bartholin cysts lined by mucinous columnar epithelium (x100; H&E stain). (**C**) Higher magnification of Bartholin cyst showing epithelium with apical mucin and basally located nuclei (×400; H&E stain).

Histopathological evaluation of the endometrial sample showed features consistent with the late secretory phase. The vulvar cyst was lined by mucinous columnar epithelium with a fibrocollagenous wall, consistent with a vulvar mucinous cyst without atypia. The vaginal lesions also showed multiple cysts lined by mucinous columnar epithelium, consistent with Bartholin gland cysts (Figure 3A–3C).

The patient had an uneventful postoperative course and was discharged in satisfactory condition. She was advised to return for follow-up after her next menstrual cycle.

## DISCUSSION

In this case, a 36-year-old multiparous woman presented with a soft, pedunculated, asymptomatic mass measuring 3 × 4 cm on the left labia majora. The lesion was unusually large for a typical vulvar cyst and was located in an atypical position—too anterior for a Bartholin gland cyst, too lateral and posterior for a Skene gland cyst, and too far lateral for a vestibular mucinous cyst. Additionally, multiple smaller, non-tender cystic swellings were noted along the inner surface of the left labia minora. All lesions were surgically excised.

Histopathological examination confirmed the labial mass to be a mucinous vulvar cyst, while the smaller lesions were consistent with multiple Bartholin gland cysts. The vulvar mass lacked the bluish hue characteristic of hemangiomas, was too fluctuant to suggest a lipoma, and exhibited a pedunculated morphology reminiscent of a giant angiomyxoma or a large fibroepithelial polyp of the vulva [8, 9].

Bartholin’s glands, typically located at the 5 and 7 o’clock positions in the vestibular groove between the hymen and labia minora, become functionally active at puberty. They measure approximately 0.5 cm and drain via 2 cm ducts [10]. Their primary role is to secrete alkaline mucus that provides lubrication during sexual arousal [10]. When the distal portion of the duct becomes obstructed, it can lead to the accumulation of glandular secretions, resulting in the formation of a Bartholin’s cyst or, if infected, an abscess [2].

When evaluating benign vulvo-vaginal masses in this anatomical location, a broad range of cystic lesions should be considered in the differential diagnosis. These include mucous cysts, cysts of the canal of Nuck, Bartholin’s duct cysts, Skene’s duct cysts, epidermal inclusion cysts, and lipogenic tumors such as lipomas and adenolipomas. Less commonly, endometriomas, post-traumatic hematomas, inguinal hernias, and vulvar syringomas may present similarly. Although rare, malignant tumors such as vulvar liposarcomas should also be kept in mind [11, 12].

Table 1 summarizes the differential diagnosis of vulvovaginal lesions, including characteristic clinical features, histopathological findings, and management strategies [11, 13–37]. These vulvovaginal lesions are benign and typically require no follow-up unless they recur.

**Table 1:** Differential diagnosis of vulvovaginal lesions - characteristic clinical features, histopathological findings, and management strategies

Vulvo-vaginal lesions	Characteristic feature	Histopathological findings	Treatment and Follow-up	Recurrence and malignant transformation	References
**Embryonic origin**					
Bartholin gland cyst	Small cystic or palpable mass in the posterior vulvar vestibule (5 and 7 o’clock) Results from blockage of the Bartholin glands due to inflammation, childbirth, trauma, or infection. Often presents as painful swelling.	Lined by residual mucinous epithelium, low cuboidal or transitional epithelium, and may show focal squamous metaplasia or denudation.	Conservative treatment with antibiotics, Simple incision and drainage, Fistulisation, Marsupialization, Complete gland excision	*Recurrence rate:* ≈10% *Malignant Transformation:* Very rare, accounts for <5% of vulvar neoplasms.	[11, 13–15]
Gartner’s duct cyst	Small cystic swelling or palpable mass along the anterolateral vaginal wall at 11 and 1 o’clock positions. Often, asymptomatic	Lined by a single layer of cuboidal to low columnar non-mucinous epithelium.	Reassurance Larger cysts may require excision.	*Recurrent rate*: ≈11.4% after vaginal excision. *Malignant Transformation:* Exceedingly rare (Very few cases reported in literature).	[11, 13, 16, 17]
Müllerian duct cyst	Typically located in the vulvar vestibule but may arise anywhere in the vagina. Results from incomplete replacement of Müllerian epithelium by squamous epithelium of the urogenital sinus.	Lined by mucin-secreting or ciliated (tubal type) columnar cells. May exhibit focal squamous metaplasia.	Reassurance Larger cysts may require excision.	*Recurrence rate:* Unknown Malignant Transformation: Very rare.	[11, 13, 18, 19]
Canal of Nuck cyst	Painless, reducible, inguinal-labial swelling. Presents as a hydrocele protruding through the inguinal canal. Appears as translucent lump over labia. Worsens on standing	Lined by Mesothelial cells.	Surgical excision and closure of the persistent canal	*Recurrence rate:* Unknown *Malignant Transformation*: Very rare, but the canal of Nuck can harbor primary malignancies like endometrioid carcinoma, Müllerian carcinosarcoma, or pseudomyxoma peritonei.	[11, 13, 20]
Skene’s duct cyst/ Urothelial cysts	Small cystic swelling or palpable mass adjacent to the urethral meatus. Derived from periurethral and Skene glands	Lined by transitional or squamous epithelium.	Recurrent or non-resolving cyst may require marsupialization.	*Recurrence rate*: Varies with the surgical approach. *Malignant Transformation:* Extremely rare.	[11, 13, 21]
**Non-embryonic lesions**					
Epidermal/inclusion cyst	Firm, round, yellow-white papulonodules Often occur after trauma or post-surgery.	Lined by keratinized stratified squamous epithelium with a preserved granular layer and filled with concentric laminated keratin.	Reassurance Incision and drainage if infected Intralesional steroids if inflamed Excision	*Recurrence rate*: Around 3% *Malignant Transformation*: Very rare (0.04%).	[11, 22, 23]
Lipoma	Soft, slow-growing subcutaneous nodule Often asymptomatic	Well-demarcated cluster of mature adipocytes	Excision	*Recurrence rate*: Complete surgical removal is rarely associated with recurrence. *Malignant Transformation*: Extremely rare.	[11, 24, 25]
Milia	Discrete, waxy papules 1–3 mm in size, dome-shaped and white to flesh-toned.	Uniform eosinophilic colloid material deposited in the papillary dermis	Reassurance Electrodessication or expression of keratin contents after incision for cosmetic purposes.	*Recurrence rate*: Unknown *Malignant Transformation*: Do not undergo malignant transformation.	[26]
Endometriosis	Tender, reddish-brown papules or nodules. Associated with cyclical pain in association with menses.	Cystic endometrial-type glands surrounded by endometrial stroma and hemosiderin-laden macrophages.	Wide local excision	*Recurrence rate:* High (6-67%) *Malignant Transformation*: Exceedingly rare.	[27, 28]
Leiomyoma	Solitary, flesh-colored to reddish-brown nodule. Mostly asymptomatic but occasionally painful	Neoplastic proliferation of smooth muscle fibers	Excision	*Recurrence rate*: Complete surgical excision is rarely associated with recurrences. *Malignant Transformation*: <1%	[29, 30]
Vaginitis emphysematosa	Variably sized vaginal nodules that produce a characteristic popping sound Very rarely reported Usually associated with a Trichomonas or Gardnerella infection	Pink colored cysts containing hyaline-like material, foreign body giant cells, and chronic inflammatory infiltrate	Reassurance Antibiotic treatment with Metronidazole	*Recurrence rate:* Self-limiting, rarely shows recurrences. *Malignant Transformation:* Does not undergo malignant transformation.	[31, 32]
Fibroepithelial stromal polyp	Benign solitary vaginal lesion of color same as patient’s skin Pedunculated	Lined by mesenchymal cells	Excision	*Recurrence rate:* Recurrence common, when not excised completely. *Malignant Transformation:* Unknown	[33]
**Lympho-vascular lesions**					
Lymphangioma	Clustered, translucent vesicles resembling “frog eggs”	Dilated lymphatic channels with flat endothelium	Reassurance Recurrence common after surgical excision, laser, or sclerotherapy	*Recurrence rate:* 23.1% *Malignant Transformation*: Very rare	[34, 35]
Angiokeratoma	Dark-red to purple papules 2–5 mm in diameter	Prominent dilated vessels in the superficial dermis beneath hyperkeratotic epidermis.	Reassurance Electrocautery or pulsed dye laser may be used for cosmetic improvement.	*Recurrence rate:* Extremely rare, as the condition itself is rare over the vulva. *Malignant Transformation:* Not known in literature.	[36, 37]

A similar case involved a 29-year-old nulligravida who presented with a slowly enlarging left vulvar mass over one year, which had recently become erythematous, painful, and tender. She reported no urinary, gastrointestinal, or gynecological symptoms. The lesion, located on the left lateral vulva, was atypical for a Bartholin gland cyst. Due to increasing discomfort, surgical excision was performed, and histopathological examination confirmed a vulvar mucinous cyst [3]. In another case, a 35-year-old woman presented with a six-year history of vaginal mass and white discharge, along with the recent onset of dysuria (6 months) and dyspareunia (3 months). She had no relevant medical or surgical history. On pelvic examination, second-degree uterovaginal prolapse was noted, along with a 5 × 4 cm cystic, non-tender mass on the left anterior and lateral vaginal wall at the mid-vaginal level. The cyst was excised, and histopathology confirmed a benign mucinous cyst [38]. In yet another case, a 12-year-old girl presented with a recurrent left vulvar cyst, initially excised 20 months prior and diagnosed as a benign Müllerian cyst. She reported discomfort during activities like cycling and horseback riding, with occasional spontaneous pain. Examination revealed a 1.5 cm translucent cyst on the posterior left labia minora. Due to persistent symptoms, surgical excision was performed. Histopathology confirmed a non-mucinous vulvar cyst lined by ciliated cuboidal to low columnar epithelium [39].

Accurate diagnosis is typically established through a combination of detailed clinical history, thorough physical examination, and radiologic imaging when necessary [4]. Cystic lesions of the vagina and vulva are typically benign and asymptomatic, and often do not require intervention. However, in select cases, complete surgical excision may be indicated, irrespective of lesion size. This approach not only facilitates a definitive histopathological diagnosis but also helps prevent potential complications such as rupture, hematoma formation, secondary infection, and, in rare instances, malignant transformation [4, 11]. Histopathological evaluation plays a critical role in differentiating these cystic lesions from other vulvar pathologies. Total excision remains a reliable and effective therapeutic strategy, particularly for large, symptomatic, or diagnostically uncertain cysts [4, 11, 12].

Bartholin’s cysts can be managed through a range of treatment options, including antibiotics, simple incision and drainage, fistulization, marsupialization, or complete gland excision [40]. Among these, surgical drainage combined with marsupialization is often the preferred approach, as it preserves glandular function and reduces the risk of recurrence or abscess formation [40]. In cases involving large or atypical cysts, total surgical excision is considered effective [2, 10].

## CONCLUSIONS

This case highlights the rare occurrence of a vulvar mucinous cyst mimicking a lipoma, coexisting with multiple Bartholin cysts in an asymptomatic reproductive-aged woman. Although vulvovaginal cysts are typically benign and silent, atypical presentations require thorough clinical assessment and surgical management. Complete excision allowed for definitive diagnosis and effective treatment, preventing potential complications. This case underscores the importance of considering rare vulvar cysts in the differential diagnosis, even in asymptomatic patients, and tailoring treatment accordingly.
